# The Mixture of Bisphenol-A and Its Substitutes Bisphenol-S and Bisphenol-F Exerts Obesogenic Activity on Human Adipose-Derived Stem Cells

**DOI:** 10.3390/toxics10060287

**Published:** 2022-05-27

**Authors:** Iris Reina-Pérez, Alicia Olivas-Martínez, Vicente Mustieles, Elena Salamanca-Fernández, José Manuel Molina-Molina, Nicolás Olea, Mariana F. Fernández

**Affiliations:** 1 Centre for Biomedical Research & School of Medicine, Radiology and Physical Medicine Department, University of Granada, 18011 Granada, Spain; irisreina@ugr.es (I.R.-P.); aolivas@ugr.es (A.O.-M.); vmustieles@ugr.es (V.M.); esalamanca@ugr.es (E.S.-F.); molinajm@ugr.es (J.M.M.-M.); nolea@ugr.es (N.O.); 2 Instituto de Investigación Biosanitaria (ibs.GRANADA), 18012 Granada, Spain; 3 CIBER de Epidemiología y Salud Pública (CIBERESP), 28029 Madrid, Spain

**Keywords:** mixtures, bisphenols, bisphenol A (BPA), bisphenol S (BPS), bisphenol F (BPF), endocrine disruptors, dose addition

## Abstract

Bisphenol A (BPA) and its substitutes, bisphenol F (BPF) and S (BPS), have previously shown in vitro obesogenic activity. This study was designed to investigate their combined effect on the adipogenic differentiation of human adipose-derived stem cells (hASCs). Cells were exposed for 14 days to an equimolar mixture of bisphenols (MIX) (range 10 nM–10 µM). Oil Red staining was used to measure intracellular lipid accumulation, quantitative real-time polymerase chain reaction (qRT-PCR) to study gene expression of adipogenic markers (PPARγ, C/EBPα, LPL, and FABP4), and Western Blot to determine their corresponding proteins. The MIX promoted intracellular lipid accumulation in a dose-dependent manner with a maximal response at 10 µM. Co-incubation with pure antiestrogen (ICI 182,780) inhibited lipid accumulation, suggesting that the effect was mediated by the estrogen receptor. The MIX also significantly altered the expression of PPARγ, C/EBPα, LPL, and FABP4 markers, observing a non-monotonic (U-shaped) dose-response, with maximal gene expression at 10 nM and 10 µM and lesser expression at 1 µM. This pattern was not observed when bisphenols were tested individually. Exposure to MIX (1–10 µM) also increased all encoded proteins except for FABP4, which showed no changes. Evaluation of the combined effect of relevant chemical mixtures is needed rather than single chemical testing.

## 1. Introduction

Evidence of the contribution of excessive and/or dysfunctional adipose tissue to the development of obesity-related diseases, including metabolic syndrome and cancer [1], has prompted a rapid increase in research on adipose tissue function. Adipocytes are key regulators of whole-body energy homeostasis, and numerous novel regulators of adipose tissue differentiation and function have been identified [2,3,4,5]. The morphology of adipose tissue is defined by the number and size distribution of its adipocytes. Tissue with numerous small adipocytes is associated with a hyperplastic morphology, and tissue with a small number of large adipocytes is associated with a hypertrophic morphology [2,6]. Increased adipocyte size in both visceral and subcutaneous adipose tissue has been associated with insulin resistance, diabetes, and cardiovascular disease, among other adverse effects [6].

Adipose tissue both stores lipids and acts as an endocrine organ that synthesises and secretes hormones; however, it can also accumulate lipophilic environmental chemical compounds known as endocrine disruptors [2,5,7,8,9]. Endocrine-disrupting chemicals (EDCs) are exogenous substances that interact with endogenous hormones at multiple levels; a) interacting, activating, and antagonising hormonal receptors; b) altering hormone receptor expression and signal transduction in hormone-responsive cells; c) inducing epigenetic modifications in hormone-producing or hormone-responsive cells; and d) altering the synthesis of hormones, their transport across cell membranes, their distribution and/or circulating levels, their metabolism or clearance, and the fate of hormone-producing or hormone-responsive cells [10,11]. There is increasing evidence of an association between exposure to some EDCs, designated as obesogens, and obesity-related diseases. These obesogens include both persistent organic pollutants (POPs) and non-persistent compounds such as bisphenols [12,13,14]. EDCs interact with multiple nuclear hormone receptors, producing an imbalance in the hormonal system [5]. Adipocyte physiology is regulated by nuclear hormone receptors, and several of these have emerged as molecular targets of various EDCs [4].

Bisphenol A (BPA), a well-known EDC, is frequently detected in both the environment and humans [15]. However, its prohibition in multiple products in 2011, when the first European BPA restrictions became effective [16], led manufacturers to start replacing BPA with its analogues [e.g., bisphenol F (BPF), bisphenol S (BPS)] in similar structure applications (thermoplastics, polycarbonate plastics, and epoxy resins), increasing human exposure to these substitute chemicals. For example, a recent European study showed that the average cumulative exposure to unconjugated BPA had decreased from 3.8 ng/kg bw/day to 2.1 ng/kg bw/day before and after the restrictions in 2011 [17]. However, despite the increased detection of BPF and BPS, they have not yet reached the levels of BPA still found in human biological samples because utilisation of the latter has not been totally prohibited, among other reasons [17,18,19,20,21].

Adverse health impacts of environmental chemicals are still assessed substance-by-substance, neglecting, for example, that humans are simultaneously exposed to mixtures of bisphenols. Evaluating their combined exposure effects is difficult but needed, and in vitro methods are potential tools enabling a better understanding of the underlying mechanisms of mixture effect [22].

In vitro and in vivo studies have supported the so-called “something from nothing” effect, i.e., the significant effect of mixtures of chemicals that are individually at a non-detectable or “no-observed effects concentration” (NOEC) [23,24,25,26,27,28]. Most research on mixtures of EDCs has addressed their activity on nuclear receptors (mainly estrogenic and androgenic receptors) and their toxic reproductive and developmental effects using in vivo models [25,29,30,31,32,33]. However, there have been scant in vivo or in vitro studies of the adipogenic activity of ad hoc mixtures of EDCs [34,35,36].

One of the most studied mechanisms of action, and for which a greater number of scientific studies are available, is related to the affinity and agonist (and/or antagonist) activity of bisphenols, BPA (to a greater extent), and its analogues BPF and BPS (to a lesser extent), on the nuclear estrogen receptors ERα and ERβ [12,17,25,37,38,39,40,41,42].

Our group recently demonstrated the effects of individual exposure to BPF and BPS on the adipogenesis and lipid metabolism of human adipose tissue-derived stem cells (hASCs) and on the expression of related genes and proteins [43]. We hypothesised that different mechanisms of action might cause interactions during combined exposure, which could lead to unpredictable effects of combined exposure [44]. The objective of the present investigation was to use a similar approach to study the ternary mixture of BPA, BPF, and BPS and determine their combined in vitro effect on adipogenic differentiation in hASCs. A secondary objective was to evaluate the role of estrogenic pathways in the action mechanism of the ad hoc bisphenol mixture.

## 2. Materials and Methods

### 2.1. Cell Culture

A commercial hASC cell line isolated from a single healthy non-diabetic adult through subcutaneous lipoaspirate, collected during elective surgical liposuction procedures (Poietics™ Normal Human ADSCs, PT-5006, Lot 0F4505, Lonza, Switzerland), was cultured and expanded following the manufacturer’s recommendations under previously reported conditions [43,45]. In total, six hASC vials with the same reference and lot number were used to carry out all experiments. In brief, cells were seeded, incubated, and expanded in growth medium (GM) at 37 °C in a humidified atmosphere containing 5% CO_2_. The GM comprised Advanced-Dulbecco’s Modified Eagle Medium (advanced-DMEM) supplemented with 10% fetal bovine serum (FBS), 2 mM GlutaMAX, 100 U mL^−1^ penicillin, and 100 µg/mL streptomycin. The culture medium was replaced every 2–3 days, and cells were passaged up to 6 times. Cells were subcultured using a mixture of 0.25% trypsin-EDTA. All products were supplied by Thermo Fisher Scientific (Gibco, Thermo Fisher Scientific, Waltham, MA, USA).

### 2.2. Adipogenic Differentiation

Differentiation was induced as previously described by Reina-Pérez et al. [43]. Briefly, hASCs were seeded in 24-well plates at initial concentrations of 40,000 cells per well in GM. When confluence was reached (2 days), the GM was replaced with a differentiation medium (DM) consisting of GM supplemented with 1 µM dexamethasone (DEX), 0.5 mM 3-isobutyl-1-methylxanthine (IBMX), 1.7 µM human insulin, and 0.1% dimethyl sulfoxide (DMSO) (vehicle) as negative control, or by the DM plus 1 µM rosiglitazone (ROSI) as positive control. Individual BPA, BPF, BPS, and the mixture of the 3 compounds (MIX) at a ratio of 1:1:1, were tested. All individual bisphenols dissolved in DMSO were mixed to produce a standard stock solution at different concentrations (0.01, 0.1, 1, and 10 mM) (Table S1). The final DMSO concentration in both the individual compounds and the mixture never exceeded 0.1% (*v*/*v*) of the culture medium [46]. All experimental conditions were finally diluted in DM and tested at concentrations of 0.01, 0.1, 1, and 10 µM during adipogenic differentiation of hASCs for 14 days [47].

hASCs were also cultured in DM containing individual bisphenols and MIX at 0.01, 0.1, 1, and 10 µM in the presence of the ER antagonist ICI 182,780 (100 nM). The DM was replaced every 2–3 days during the adipogenic process. All differentiation products were supplied by Sigma (Sigma-Aldrich, St. Louis, MO, USA) except for ICI 182,780 (Tocris Bioscience, Bristol, UK).

### 2.3. Quantitative Oil Red O Staining Assay

At 14 days of adipogenic differentiation, Oil Red O (ORO) staining was performed to quantify the accumulation of intracellular lipids, mainly triglycerides, in mature adipocytes obtained from all controls and under different experimental conditions (0.1, 1, and 10 µM with or without ICI 182,780 at 100 nM). Briefly, cells were washed with phosphate-buffered saline (PBS) and fixed in 4% paraformaldehyde (Electron Microscopy Science Hartfield, PA, USA) for 1 h at room temperature. After washing with milliQ-water and 60% isopropanol, cells were stained with a filtered ORO solution (0.5%, *w*/*v*) in milliQ- water (60/40, *v*/*v*) for 45 min, followed by washing with 60% isopropanol and again with milliQ-water. Cells were first observed and photographed under a Leica DMi8 microscope (Leica Microsystems, Wetzlar, Germany) with the Leica Application Suite (LAS) X software, and the retained dye was extracted with 100% isopropanol, measuring the optical density at a wavelength of 520 nm with a microplate reader (BioTek HTX, Fisher Scientific, Waltham, MA, USA) [48]. All aforementioned products were purchased from Thermo Fisher Scientific (Gibco, Thermo Fisher Scientific, Waltham, MA, USA) or Sigma Aldrich (Sigma-Aldrich, St. Louis, MO, USA).

### 2.4. RNA Isolation and Quantitative Real-Time Polymerase Chain Reaction (qRT-PCR)

On day 14 of adipogenic differentiation, total RNA was extracted from cells using the RNeasy Mini kit supplied by Qiagen (Qiagen, Hilden, Germany) according to the manufacturer’s instructions. The RNase-Free DNase kit supplied by Qiagen (Qiagen, Hilden, Germany) was used to eliminate genomic DNA, following the manufacturer’s instructions. The final RNA concentration and quality (260/280 ratio) were determined with a Nanodrop 2000 spectrophotometer (Thermo Fisher Scientific, Waltham, MA, USA). The iScript cDNA Synthesis Kit (Bio-Rad Laboratories, Hercules, CA, USA) was used to transcribe 1000 ng of total RNA into cDNA, following the manufacturer’s instructions.

RT-qPCR was used to measure the expression levels of genes involved in the adipogenic process, including peroxisome proliferator-activated receptor gamma (PPARγ), CCAT/enhancer-binding protein (C/EBPα), lipoprotein-lipase (LPL), and fatty acid-binding protein 4 (FABP4). Hypoxanthine-guanine phosphoribosyltransferase-1 (HPRT1) and β-actin (ACTB) served as control or housekeeping genes for all experiments. Primer pairs for each target gene were PPARγ (Assay ID qHsaCED0044425), C/EBPα (Assay ID qHsaCED0019045), LPL (Assay ID qHsaCED0047106), FABP4 (Assay ID qHsaCED0057474), HPRT1 (Assay ID qHsaCID0016375), and ACTB (Assay ID qhsaLED0214042), all acquired from Bio-Rad (Bio-Rad Laboratories, Hercules, CA, USA). RT-qPCR was carried out with an ABI Prism 7900HT instrument using SYBR Green PCR (Bio-Rad Laboratories, Hercules, CA, USA). The Qiagen Data Analysis Center (GeneGlobe Data Analysis Center, Qiagen, Hilden, Germany) was used for the quantification. The stability of reference genes was statistically validated for each sample in duplicate, employing the 2^−ΔΔCt^ method to express the results as fold-changes and using the negative control as reference [49].

### 2.5. Western Blot

At 14 days of adipogenic differentiation, protein levels of selected genes were measured in cells cultured under the different experimental conditions (0.01, 0.1, 1, and 10 µM MIX). Briefly, lysis was performed using cell lysis buffer [10 mM Tris-HCl pH 7.5, 150 mM NaCl, 2 mM EDTA, 1% Triton X-100, 100% glycerol (Sigma-Aldrich, St. Louis, MO, USA)], a protease inhibitor cocktail (Thermo Fisher Scientific, Waltham, MA, USA), and β-mercaptoethanol (Sigma-Aldrich, St. Louis, MO, USA), and samples were then placed on ice for 20 min [36]. After centrifugation for 30 min at 13,000× *g* and 4 °C, the protein content of the supernatant was quantified by DC Protein Assay, using 50 µg of protein sample mixed with 4X Laemmli sample buffer containing 10% β-mercaptoethanol and milliQ-water. Samples were separated with SDS-PAGE using TGX Any kD gel and transferred onto a nitrocellulose membrane, which was incubated in blocking buffer [5% non-fat milk in 1X Tris-buffered saline (TBS) with 0.5% Tween 20 (TBS-T)] for 1 h at room temperature. Primary antibodies to incubate blotted membrane for PPARγ, C/EBPα, FABP4, LPL, and HSC-70 (B6) were used with appropriate horseradish peroxidase-labelled secondary antibodies (Table S2). Immunoreactive signals were detected using the Clarity Western ECL Substrate Kit, and membranes were digitally imaged with Image Reader LAS-4000 and quantified by densitometry using ImageJ software. Protein levels were represented as the fold-changes in expression relative to the control (HSC-70). All these products were acquired from Bio-Rad (Bio-Rad Laboratories, Hercules, CA, USA) or Sigma Aldrich (Sigma-Aldrich, St. Louis, MO, USA).

### 2.6. Cell Viability

The number of viable cells was determined using the trypan blue test described by Strober with slight modifications [50]. Briefly, after 14 days of culture under the aforementioned experimental conditions (0.01, 0.1, 1, and 10 µM of MIX), cells were dissociated with trypsin, resuspended in PBS, mixed with trypan blue dye, visually examined, and counted in a Neubauer chamber to determine the percentage of viability under each experimental condition. An Olympus IX51 inverted microscope with Olympus TH4-200 lamp was used.

### 2.7. Statistical Analysis

Cell cultures were repeated at least three times for each condition with multiple replicates. Analyses of gene and protein expression were performed in duplicate for each sample. The viability and ORO staining assays were carried out with multiple replicates for each experimental condition. Data were expressed as means ± standard error of the mean (SEM). Significant differences in assay results were evaluated using the non-parametric Mann–Whitney U test. A ≥1.5-fold increase and decrease in gene expression was considered as up-regulation and down-regulation, respectively. SPSS version 23 (IBM SPSS, Armonk, NY, USA) was used for statistical analyses, considering * *p* < 0.05 as significant.

## 3. Results

### 3.1. The Bisphenol Mixture Promotes Intracellular Lipid Accumulation in a Dose-Dependent Manner

**Figure 1:**
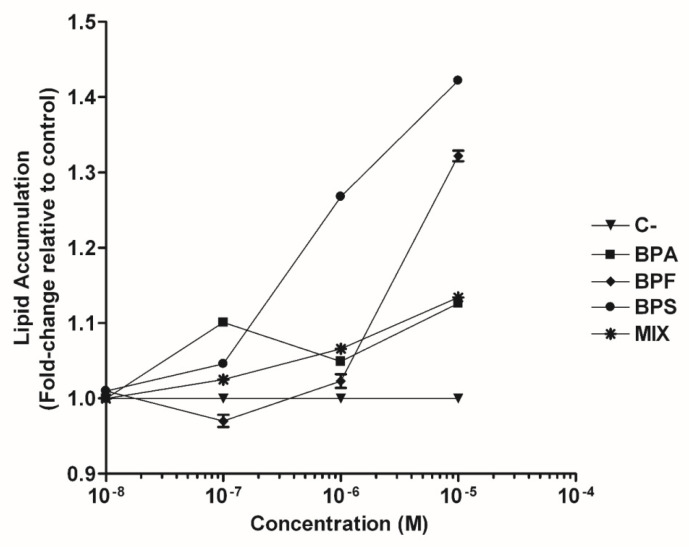
Effect of BPA, BPS, BPF, or a mixture of three bisphenols (BPA, BPS, and BPF) on intracellular lipid accumulation in human adipose-derived stem cells (hASCs). Lipid accumulation was quantified by Oil Red O staining assay (at 520 nm) after 14 days in the presence of 0.01, 0.1, 1, or 10 μM. Lipid content was normalised using the negative control and expressed as fold-changes. Data were expressed as means ± SEM from three independent experiments with multiple replicates for each experimental condition. BPA, bisphenol A; BPF, bisphenol F; BPS, bisphenol S; MIX (BPA, BPF, and BPS); C−, negative control.

hASCs were differentiated into mature adipocytes under the following experimental conditions for 14 days: negative control (0.1% DMSO-vehicle), positive control (1 µM ROSI), BPA, BPS, BPF, or bisphenol mixture at concentrations of 0.01, 0.1, 1, or 10 µM. Adipogenic differentiation was confirmed morphologically by the accumulation of intracellular lipid droplets. A dose-dependent increase in intracellular lipid accumulation of mature adipocytes was observed, with a maximum response at 10 µM but with different potencies, in the order BPS (1.422 ± 0.004) > BPF (1.322 ± 0.007) > MIX (1.134 ± 0.005) > BPA (1.126 ± 0.005) (Figure 1 and Figure S1, Table S3).

**Figure 2:**
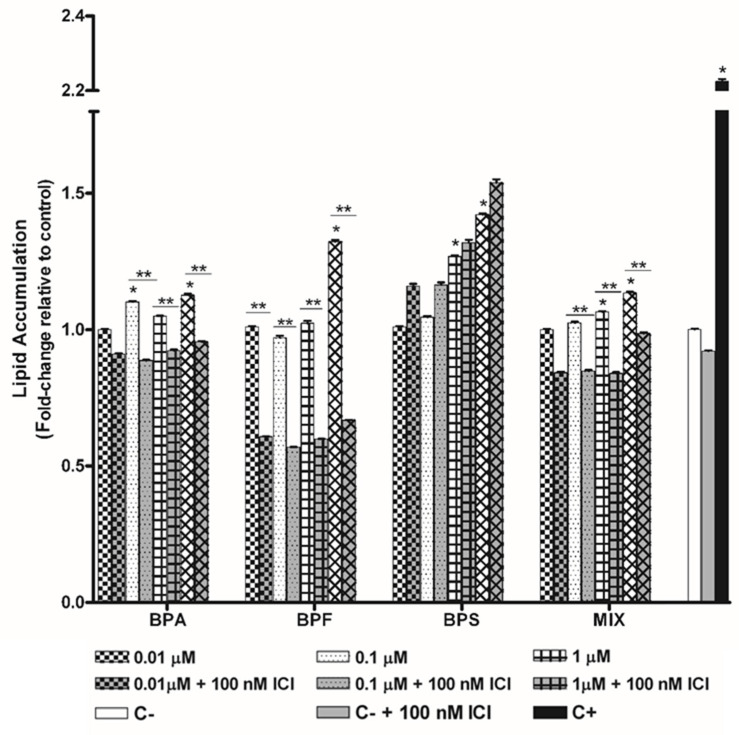
Lipid accumulation in mature adipocytes was quantified by ORO staining assay (absorbance at 520 nm) after 14 days of hASCs differentiation in the presence of BPA, BPS, BPF, or the mixture, with or without 100 nM ICI 182,780. Lipid content was normalised using the negative control and expressed as fold changes. Data were expressed as means ± SEM from three independent experiments with multiple replicates for each experimental condition. Significant differences were analysed using the Mann–Whitney U test and defined as * *p* < 0.05 respect to negative control, and ** *p* < 0.05 respect to the experimental condition without ICI. BPA, bisphenol A; BPF, bisphenol F; BPS, bisphenol S; MIX (BPA, BPF, BPS); C−, negative control.

hASCs were also treated under the same experimental conditions in the presence of the antiestrogen ICI 182,780 at a fixed concentration of 100 nM. Lipid accumulation was also studied for both individual bisphenols and the bisphenol mixture. The intracellular lipid accumulation of mature adipocytes (by ORO staining assay) was significantly reduced at most of all tested concentrations of the bisphenol mixture (15%, 17.3%, 21.2%, and 13% at 0.01, 0.1, 1, and 10 µM, respectively; range: 79–87%) (Figure 2, Table S3), finding lipid accumulation inhibition ranging from 80 to 88%. Similar results were found for BPA, with higher inhibition rates (50 to 60%) with BPF but no inhibition with BPS, as demonstrated by ORO results for mature adipocytes (Figure 2, Table S3). Treatment with ICI alone did not significantly affect lipid accumulation (data not shown).

### 3.2. The Bisphenol Mixture Alters the Expression of Adipogenesis-Related Genes in a Non-Monotonic Manner

**Figure 3:**
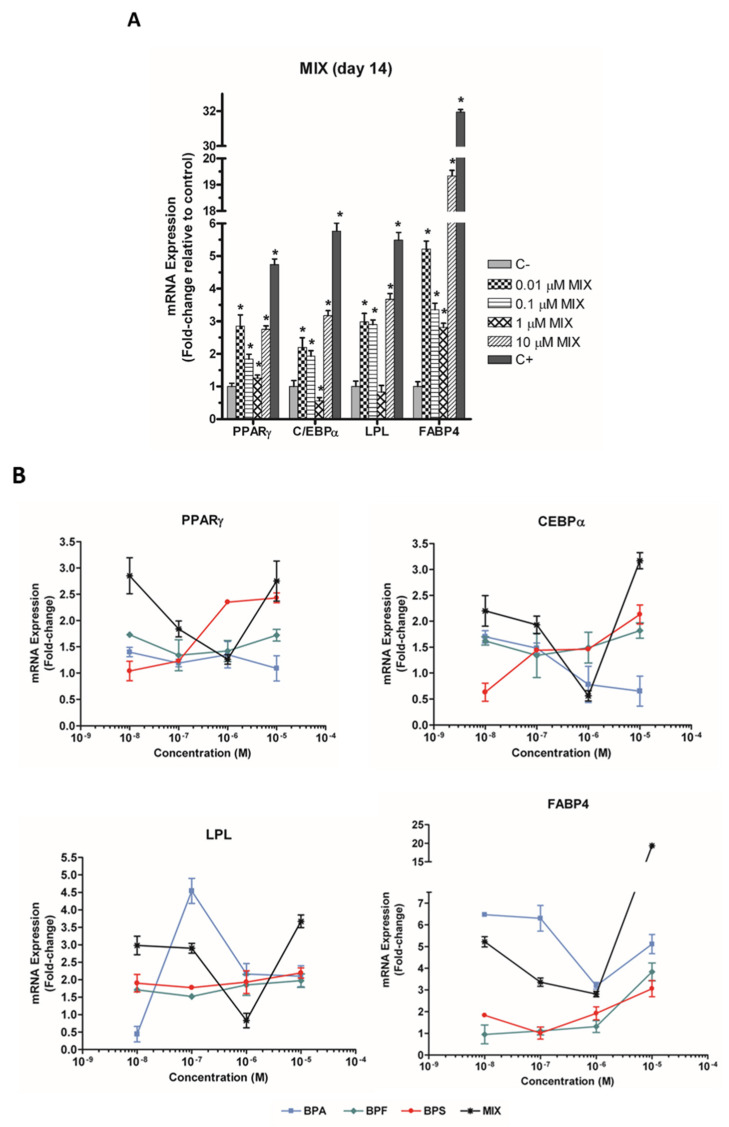
(**A**) Effect of the mixture of three bisphenols (BPA, BPS, and BPF), and (**B**) effect of individual compounds, on the expression of the adipogenic marker genes PPARγ, C/EBPα, LPL, and FABP4 at 14 days of adipogenic differentiation in hASC cultures. mRNA levels were normalised using the levels of control genes (HPRT1 and ACTB) and expressed as fold changes by the 2^−ΔΔCt^ method. Data were expressed as means ± SEM of three independent experiments with multiple replicates for each condition. Significant differences were analysed using the Mann–Whitney U test and defined as * *p* < 0.05. ACTB, β-actin; C/EBPα, CCAT/enhancer-binding protein; FABP4, fatty acid-binding protein 4; HPRT1, hypoxanthine-guanine phosphoribosyltransferase-1; LPL, lipoprotein-lipase; PPARγ, peroxisome proliferator-activated receptor gamma; MIX (BPA, BPF and BPS); C−, negative control; C+, positive control.

hASC treatment with the bisphenol MIX (0.01, 0.1, 1, and 10 µM) significantly increased the mRNA expression of PPARγ, C/EBPα, LPL, and FABP4 genes, observing a non-monotonic dose-response in all cases. In this way, PPARγ mRNA expression was increased 2.85-fold at 0.01 µM, 1.26-fold at 1 µM, and 2.75-fold at 10 µM (Figure 3A). C/EBPα and LPL expressions showed the same pattern (2.2-, 1.93-, 0.56-, and 3.17-fold, and 2.98-, 2.90-, 0.83, and 3.67-fold, respectively, at 0.01, 0.1, 1, and 10 µM MIX) (Figure 3A). The highest increase was in FABP4 mRNA expression (5.22-, 3.36-, 2.81-, and 19.34-fold, at 0.01, 0.1, 1, and 10 µM of MIX, respectively) (Figure 3A).

The same genes were evaluated on day 14 in the presence of BPA, BPF, or BPS. Results indicate that the effect of the mixture differs from those of individual components (Figure 3B, Table S4).

### 3.3. The Bisphenol Mixture Alters Protein Levels of Selected Adipogenic Markers

**Figure 4:**
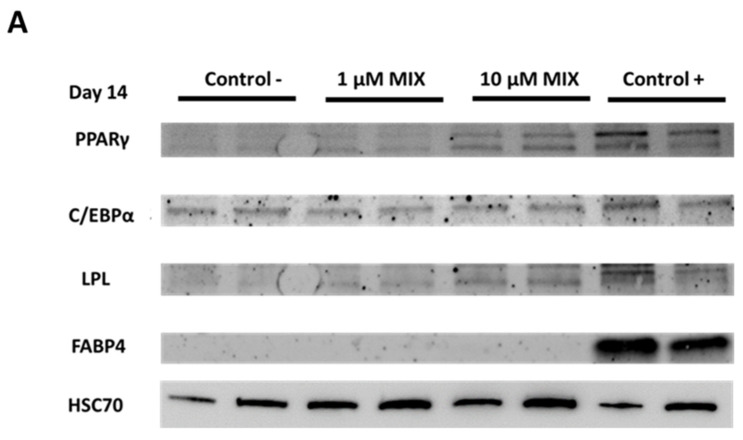

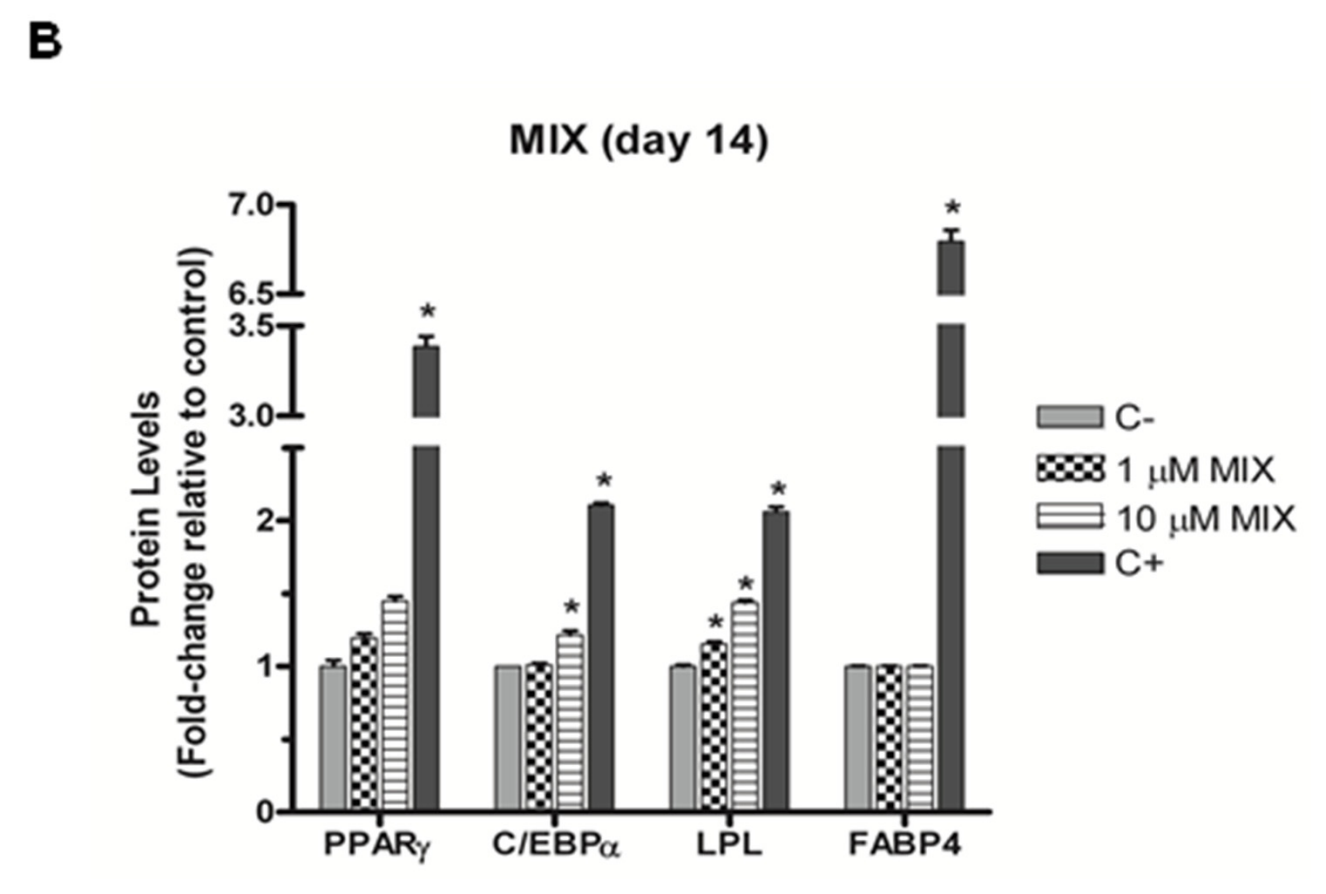
Effect of a mixture of three bisphenols (BPA, BPS, and BPF) on the protein expression of the adipogenic markers PPARγ, C/EBPα, LPL, and FABP4. Protein levels were determined by Western Blot (**A**) and densitometry (**B**) after 14 days of hASCs differentiation in the presence of MIX at 1 and 10 µM. Protein levels were normalised using protein control levels (HSC70) and expressed as fold changes. Data were expressed as means ± SEM of three independent experiments, performed in duplicate for each condition. Significant differences were evaluated using the Mann–Whitney U test and defined as * *p* < 0.05. C−, negative control; C+, positive control.

hASC treatment with the bisphenol MIX (0.01, 0.1, 1, and 10 µM) increased the levels of proteins encoded by PPARγ, C/EBPα, and LPL at the higher concentrations used (1 and 10 µM) (Figure 4A,B). Specifically, a slight but significant increase was observed in LPL protein levels (1.16-fold) at 1 µM MIX (Figure 4B) and in C/EBPα and LPL protein levels (1.22- and 1.44-fold, respectively) at 10 µM MIX (Figure 4B). FABP4 protein levels did not differ from negative control results at any of the tested concentrations of MIX. At the lowest MIX concentrations tested (0.01 and 0.1 µM) the control protein bands (HSC70) were detected for the conditions studied but not for selected adipogenic markers (PPARγ, C/EBPα, LPL, and FABP4) (Figure S2).

### 3.4. Effects of Bisphenols Mixture on hASC Viability

No cell cytotoxicity was observed in hASCs at any concentration (0.01, 0.1, 1, or 10 µM). At least 90% cell viability was obtained under all experimental conditions (Figure S3).

## 4. Discussion

An equimolar mixture of BPA, BPF, and BPS interfered with the differentiation programming of hASCs into adipocytes and altered their intracellular lipid accumulation, as previously described for each of these bisphenols [43,51,52,53,54]. However, our results indicated that the effect of the mixture differed from that of the individual components. Furthermore, this ternary bisphenol mixture affected the expression of genes involved in the adipogenesis process, but in a non-monotonic dose-response manner, not observed when analysing bisphenols individually. To our best knowledge, this is the first in vitro study to investigate the combined obesogenic activity of a mixture of three known endocrine disruptors (BPA and its analogues BPF and BPS) on the adipogenic differentiation, lipid accumulation, gene expression, and protein synthesis of stem cells derived from human adipose tissue.

The potential obesogenic effect of each individual bisphenol (BPA, BPF, and BPS) has been widely demonstrated in in vivo and in vitro studies [43,51,52,53,54,55,56,57]. In this way, in vitro studies have assessed the effects of these bisphenols on the adipogenic differentiation of murine 3T3-L1 cells, committed to being preadipocytes [58,59,60,61,62] and hASCs, which are of greater relevance to human adipogenesis [43,51,52,53,54,55]. Individually, all three bisphenols interfered with the developmental programming of hASCs, enhancing their differentiation into adipocytes and accumulation of intracellular lipid droplets [43,51,52,53,55,63,64]. Most of the effects shown were dose-dependent and corroborated by the up-regulation of specific adipogenic genes and their corresponding proteins [43,54]. However, there is still some heterogeneity in the responses of many metabolism-disrupting chemicals in adipogenic differentiation studies. In previous studies, binary bisphenol mixtures (BPA+BPF and BPA+BPS) were found to exert potentially synergistic cytotoxic effects on *Ctenopharyngodon Idella* kidney cells [65]. A synergistic effect was also observed for mixtures of three components of BPA and BADGE analogues [65]. Some in vitro studies revealed additive effects of binary and multicomponent mixtures of bisphenols on estrogen and androgen receptor activity [25,47]. Likewise, an in vitro study on their genotoxic activity found that mixtures of bisphenols increased the gene expression of pivotal metabolic enzymes CYP1A1 and UGT1A1 by HepG2 cells in an additive manner [30]. In this sense, Backhaus and Faust suggested that since cases of more than additive mixtures effects seem to be rare, to consider additivity as precautious first tier, regardless of the mechanisms of action of the mixture components [22].

The present study contributes new evidence on the combined effect of bisphenol mixtures, drawing attention to the potentially hazardous effects of the coexistence of certain bisphenols in the environment [31]. Interestingly, we observed that some of the effects depended on concentrations but in a paradoxical manner. For instance, the adipogenic gene expression was stimulated at both high (10 µM) and low (10 nM) MIX concentrations, but the effect was always lower at intermediate concentrations (1 µM). The concentrations tested in this study were selected because they have been used in in vitro studies of these bisphenols in human cell lines [43,63,66,67] and are considered environmentally relevant [18,19,20,21]. No in vitro data are available on human cell lines treated with similar concentration mixtures for comparisons; however, the non-monotonic U-shaped response observed for some of the genes in the present study was also described by Wang and co-workers [68].

Elucidation of the precise signalling pathways altered by exposure to environmental contaminants will help to identify and understand chemicals that pose a threat to metabolic health. In the present study, we have investigated some of the molecular pathways involved in the differentiation process, evaluating genes related to adipocyte development and lipid metabolism. The selected genes were PPARγ, the master regulator gene of adipogenesis [58,69]; C/EBPα, a mid-stage marker [69,70]; and LPL and FABP4, late markers of adipocyte maturation [69,71]. The mRNA expression pattern of each gene was also assessed. In comparison to the results recently published by our group for the individual bisphenols in the mixture at the same concentration range and under the same culture conditions [43], the gene expression was generally higher (1- to 20-fold for MIX, 1- to 4-fold for BPF, 1- to 3-fold for BPS, and 1- to 8-fold for BPA) (Figure 3A,B).

Statistically significant increases were also observed in protein levels of PPARγ, C/EBPα, and LPL at 14 days of hASCs differentiation in the presence of the mixture at concentrations of 1 and 10 µM (Figure 4). PPARγ, C/EBPα, and LPL protein levels were generally higher when treated at a concentration of 10 µM with the mixture of bisphenols (BPA, BPF, and BPS) than when treated with each individual bisphenol at the same concentration: 1- to 1.5-fold for MIX, 1- to 1.35-fold for BPF, and 1- to 1.1-fold for BPS. However, Western Blot results showed no effect on FABP4 protein levels at these concentrations, as previously reported for individual treatments with BPF and BPS at 10 µM [43], indicating perhaps that higher gene expression would be needed to obtain quantifiable levels of this protein, as shown by its positive control.

Previous studies have shown that the adipogenic effect of BPA is exerted, at least in part, through ER activation, as this effect can be blocked by the ER antagonist ICI 182,780 [15,53], as is confirmed in this work. This mode of action would therefore represent one of the underlying pathways between bisphenols exposure and obesity risk [53]. The obesogenic capacity of BPF was also inhibited by ICI at all concentrations tested but had no such effect on BPS under these study conditions, as reported [43]. Similarly, in their zebrafish reporter gene assay on the in vitro effect of individual bisphenols on ERs (ERα, ERβ1, and ERβ2), Le Fol et al. [72] also found that ICI 182,780 at 1 µM blocks the effect of BPA and BPF but not of BPS. Estrogen receptor pathways do not emerge as the main activation pathway for BPS, which could exert its obesogenic effects via PPARγ activation [51,58]. In this regard, Schaffert et al. recently investigated the mode of action of BPA and four of its substitutes during the differentiation of human preadipocytes [73]. BPA, BPS, and BPF disrupted crucial metabolic functions and insulin signalling at low and environmentally relevant concentrations, and the effects were mediated via the inhibition of PPARγ. Previously, Boucher et al. observed that BPS promotes the adipogenic differentiation of primary human preadipocytes and induces lipid accumulation via PPARγ pathway [51,52]. Overall, given that adipogenesis involves the timely expression of various key transcription factors (PPARγ, GR, C/EBPα, ER, etc.) and bisphenols have an affinity for several receptors and enzymes, it is highly likely that their obesogenic effects result from a combination of multiple pathways [41,69].

The estrogenic activity of bisphenols mixtures remains underexplored [25,47]. In this work, we investigated whether the adipogenic capacity of the bisphenols mixture could be blocked by the competitive ER antagonist ICI 182,780. According to the present results, the presence of 100 nM ICI for 14 days induced inhibition of intracellular lipid accumulation in hASCs treated with the mixture at all tested concentrations (Figure 2, Table S3). The inhibition was not concentration-dependent and differed from that found with the individual components of the mixture. In our study, the inhibitory action of ICI on the bisphenol mixture could not be predicted from in vitro data on the action of each individual bisphenol. Other authors reported that binary mixtures of BPA, BPF, and/or BPS had similar effects on various ERs [74]. It has been proposed that when individual environmental pollutants act via the same mechanism of action (e.g., ER activation), the effect of their mixture can be predicted according to a concentration addition model [25].

Study limitations include: the difficulty in identifying the molecular mechanisms underlying the impact of bisphenols on adipocyte biology; the lack of discrimination between hyperplasia and hypertrophy in mature adipocytes; and the influence on the results of the characteristics of the selected human mesenchymal stem cell line, given the wide variability according to the sex, ethnicity, and physiological status of donors (in the present case, a single donor). Furthermore, these in vitro results cannot be directly extrapolated to humans, and the proportions of BPA, BPF, and BFS in the ad hoc mixture do not necessarily reflect detectable levels in the environment. On the other hand, all hASCs studied were from the same donor, avoiding genetic variability, and the relevance of findings to humans was enhanced by using hASCs as a model of adipogenesis. Strengths also include the contribution of new evidence on the combined effect of mixtures of bisphenols, highlighting the potentially harmful impact of the co-presence of certain bisphenols and derivatives in the environment.

It is necessary to implement standardised adipogenic differentiation protocols to improve the consistency of results. Kassotis et al. recently published a comprehensive assessment of inter-laboratory reproducibility, analysing factors contributing to the variability in responses to these chemical contaminants [75]. Human mesenchymal stem cell and pre-adipocyte models require further validation to improve the translation of this knowledge to human metabolic health. Scientific knowledge can also be improved by combining traditional in vitro models with new assessment techniques such as three-dimensional and co-culture techniques that allow the analysis of environmentally relevant levels of exposure [76].

## 5. Conclusions

Humans are simultaneously exposed to complex mixtures of different bisphenols, among numerous environmental EDCs. This study sheds some light on mixtures of bisphenols’ obesogenic effects on human adipose-derived stem cells. A ternary mixture of bisphenols altered lipid accumulation and the mRNA expression of genes and proteins of adipogenesis markers at 14 days of differentiation, inducing changes that differed from the individual effects of each bisphenol. Evaluation of the combined effect of relevant EDC mixtures is needed rather than single chemicals testing, and in vitro methods allow a better understanding of the underlying molecular mechanisms. Further studies should be performed to identify the causal mechanism underlying the observed effects.

## Data Availability

Data supporting the reported results are available on request.

## Supplementary Materials

The following supporting information can be downloaded at: https://www.mdpi.com/article/10.3390/toxics10060287/s1, Figure S1: hASCs visualised by Oil Red O staining assay after 14 days of adipogenic differentiation; Figure S2: Western Blot of the control protein (HSC70) at the lowest concentrations of the mixture tested (0.01 and 0.1 µM). Figure S3: Effect of the mixture of three bisphenols (BPA, BPS, and BPF) on the viability of hASCs after 14 days of adipogenic differentiation. Table S1: Standard stock solutions of a mixture of three bisphenols (BPA, BPF, and BPS); Table S2: Brief description of primary and secondary antibodies used in Western Blot; Table S3. Intracellular lipids assessed by Oil Red O assay in hASCs after 14 days; Table S4. Gene expression of the adipogenic markers PPARγ, C/EBPα, LPL, and FABP4, in hASCs after 14 days of adipogenic differentiation.

## Abbreviations

ACTB, β-actin; advanced-DMEM, Advance-Dulbecco Modified Eagle Medium; BPA, bisphenol A, 4,4′-isopropylidenediphenol; BPF, bisphenol F, 4,4′-methylenediphenol; BPS, bisphenol S, 4,4′-sulfonyldiphenol; cDNA, complementary deoxyribonucleic acid; C/EBPα, CCAT/enhancer-binding protein; DEX, dexamethasone; DM, differentiation medium; DMSO, dimethyl sulfoxide; EDC, endocrine disrupting chemical; ER, estrogen receptor; FABP4, fatty acid binding protein 4; FBS, fetal bovine serum; GM, growth medium; hASCs, human adipose-derived stem cells; ICI, ER antagonist ICI 182,780; INS, human insulin; HPRT1, hypoxanthine-guanine phosphoribosyltransferase-1; IBMX, 3-isobutyl-1-methylxanthine; LPL, lipoprotein-lipase; MIX, bisphenols mixture; μg, micrograms; μM, micromolar; mM, millimolar; mL, millilitre; min, minutes; mRNA, messenger ribonucleic acid; ng, nanograms; nm, nanometers; C−, negative control; NOEC, no observed effects concentration; ORO, Oil Red O; PPARγ, peroxisome proliferator- activated receptor gamma; PBS, phosphate buffered saline; POPs, persistent organic pollutants; C+, positive control; qRT-PCR, quantitative real-time polymerase chain reaction; ROSI, rosiglitazone; SDS-PAGE, sodium dodecyl sulfate polyacrylamide gel electrophoresis; SEM, standard error of the mean; TBS, tris-buffered saline; TBS-T, Tris-buffered saline-Tween 20.
