# Supplementary material for: The Mixture of Bisphenol-A and Its Substitutes Bisphenol-S and Bisphenol-F Exerts Obesogenic Activity on Human Adipose-Derived Stem Cells
Source: Toxics. 2022 May 27;10(6):287. doi: 10.3390/toxics10060287 (PMC9229358; doi:10.3390/toxics10060287)
Supplement: Supplementary file 1 [file toxics-10-00287-s001.zip › toxics-1575950-supplementary.pdf]

toxics

Supplementary Materials: The Mixture of Bisphenol-A and Its Substitutes Bisphenol-S and Bisphenol-F Exerts Obesogenic Activity on Human Adipose-Derived Stem Cells

Iris Reina-Pérez, Alicia Olivas-Martínez, Vicente Mustieles, Elena Salamanca-Fernández, José Manuel Molina-Molina, Nicolás Olea and Mariana F. Fernández

Table S1. Standard stock solutions of a mixture of three bisphenols (BPA, BPF, and BPS).

Mixture of three bisphenols (MIX)	
1.5 mL of MIX 0.01 mM	500 µL of BPA 0.01 mM + 500 µL of BPF 0.01 mM + 500 µL of BPS 0.01 mM
1.5 mL of MIX 0.1 mM	500 µL of BPA 0.1 mM + 500 µL of BPF 0.1 mM + 500 µL of BPS 0.1 mM
1.5 mL of MIX 1 mM	500 µL of BPA 1 mM + 500 µL of BPF 1 mM + 500 µL of BPS 1 mM
1.5 mL of MIX 10 mM	500 µL of BPA 10 mM + 500 µL of BPF 10 mM + 500 µL of BPS 10 mM

Table S2. Brief description of primary and secondary antibodies used in Western Blot.

<table>
<tr><th colspan="2">Peptide/Protein Target</th><th>Manufacturer, Catalog number</th><th>Species</th><th>Dilution</th></tr>
<tr><td rowspan="5">Primary Antibodies</td><td>PPARγ</td><td>Cell Signaling Technology, 2435</td><td rowspan="4">Rabbit</td><td rowspan="3">1:1000 (5% BSA in 1X TBS with 0.5% Tween 20)</td></tr>
<tr><td>C/EBPα</td><td>Cell Signaling Technology, 2841</td></tr>
<tr><td>FABP4</td><td>Cell Signaling Technology, 3544</td></tr>
<tr><td>LPL</td><td>Abcam, ab172953</td><td rowspan="4">1:1000 (5% non-fat milk in 1X TBS with 0.5% Tween 20)</td></tr>
<tr><td>HSC-70 (B6)</td><td>Santa Cruz Biotechnology, SC-7298</td><td>Mouse</td></tr>
<tr><td rowspan="2">Secondary Antibodies</td><td>Goat Anti-Rabbit IgG (H+L)-HRP Conjugate</td><td>BIO-RAD, 1706515</td><td>Anti-Rabbit</td></tr>
<tr><td>Goat Anti-Mouse IgG (whole molecule)–Peroxidase Conjugate</td><td>Sigma, A4416</td><td>Anti-Mouse</td></tr>
</table>

Table S3. Intracellular lipids assessed by Oil Red O bioassay in hASCs after 14 days.

	**BPA**				**BPF**				**BPS**				**Mixture**			
	0.01 μM	0.1 μM	1 μM	10 μM	0.01 μM	0.1 μM	1 μM	10 μM	0.01 μM	0.1 μM	1 μM	10 μM	0.01 μM	0.1 μM	1 μM	10 μM
No ICI	1.050 ± 0.003	*1.101 ± 0.004	1.049 ± 0.002	*1.126 ± 0.005	1.010 ± 0.003	0.970 ± 0.008	1.023 ± 0.009	*1.322 ± 0.007	1.030 ± 0.003	1.046 ± 0.004	*1.268 ± 0.005	*1.422 ± 0.004	1.050 ± 0.003	1.025 ± 0.004	*1.066 ± 0.001	*1.134 ± 0.005
ICI	0.880 ± 0.003	*0.886 ± 0.003	0.923 ± 0.004	0.955 ± 0.003	0.560 ± 0.001	*0.569 ± 0.001	*0.599 ± 0.001	*0.667 ± 0.002	1.159 ± 0.009	1.164 ± 0.009	*1.319 ± 0.011	*1.539 ± 0.013	0.842 ± 0.004	*0.848 ± 0.004	*0.840 ± 0.004	*0.986 ± 0.004
%	88.00	80.49	87.97	84.74	55.44	58.63	58.50	50.45	114.75	111.30	104.07	108.20	84.2	82.71	78.82	86.97

Cells were exposed to BPA, BPF, BPS or the mixture of the three bisphenols. Lipid content was normalized using the negative control and expressed as fold-changes. Differences were analyzed using the Mann-Whitney U test and defined (*p<0.05). BPA, bisphenol A; BPF, bishpenol F; BPS, bisphenol S; ICI 182,780. % inhibition percentage.

Table S4. Gene expression of the adipogenic markers PPARγ, C/EBPα, LPL, and FABP4, in hASCs after 14 days of adipogenic differentiation.

	**BPA**				**BPF**				**BPS**				**Mixture**			
	0.01 μM	0.1 μM	1 μM	10 μM	0.01 μM	0.1 μM	1 μM	10 μM	0.01 μM	0.1 μM	1 μM	10 μM	0.01 μM	0.1 μM	1 μM	10 μM
PPARγ	*1.40 ±0.09	*1.19 ±0.070	*1.35 ±0.250	*1.09 ±0.240	1.73 ±0.062	1.34 ±0.295	1.42 ±0.199	*1.72 ±0.113	1.04 ±0.185	1.23 ±0.004	*2.35 ±0.008	*2.43 ±0.094	*2.85 ±0.342	*1.84 ±0.151	*1.26 ± 0.09	*2.75 ± 1.09
C/EBPα	*1.70 ±0.120	*1.48 ±0.100	0.78 ±0.350	*0.65 ±0.290	1.62 ±0.081	1.34 ±0.427	*1.49 ±0.297	*1.82 ±0.148	0.63 ±0.173	1.44 ±0.031	*1.46 ±0.037	*2.13 ±0.181	*2.2 ±0.295	*1.93 ±0.169	*0.56 ±0.100	*3.17 ±0.156
LPL	*0.44 ±0.220	*4.540 ±0.360	*2.16 ±0.300	*2.10 ±0.300	1.71 ±0.099	1.52 ±0.050	1.85 ±0.301	1.97 ±0.193	*1.90 ±0.251	1.77 ±0.041	*1.93 ±0.324	*2.19 ±0.148	*2.98 ±0.265	*2.90 ±0.140	0.83 ±0.208	*3.67 ±0.180
FABP4	*6.47 ±0.100	*6.30 ±0.590	*3.19 ±0.160	*5.11 ±0.440	0.95 ±0.431	1.11 ±0.180	1.31 ±0.274	3.83 ±0.409	1.83 ±0.118	1.01 ±0.281	1.92 ±0.296	3.06 ±0.374	*5.22 ±0.238	*3.36 ±0.193	*2.81 ±0.127	*19.34 ±0.210

Cells were exposed to BPA, BPF, BPS or the mixture of the three bisphenols. Data were expressed as fold-changes ± SEM of three independent experiments with multiple replicates for each condition. Significant differences were analyzed using the Mann-Whitney U test and defined as *$p < 0.05$. BPA, bisphenol A; BPF, bishpenol F; BPS, bisphenol S.

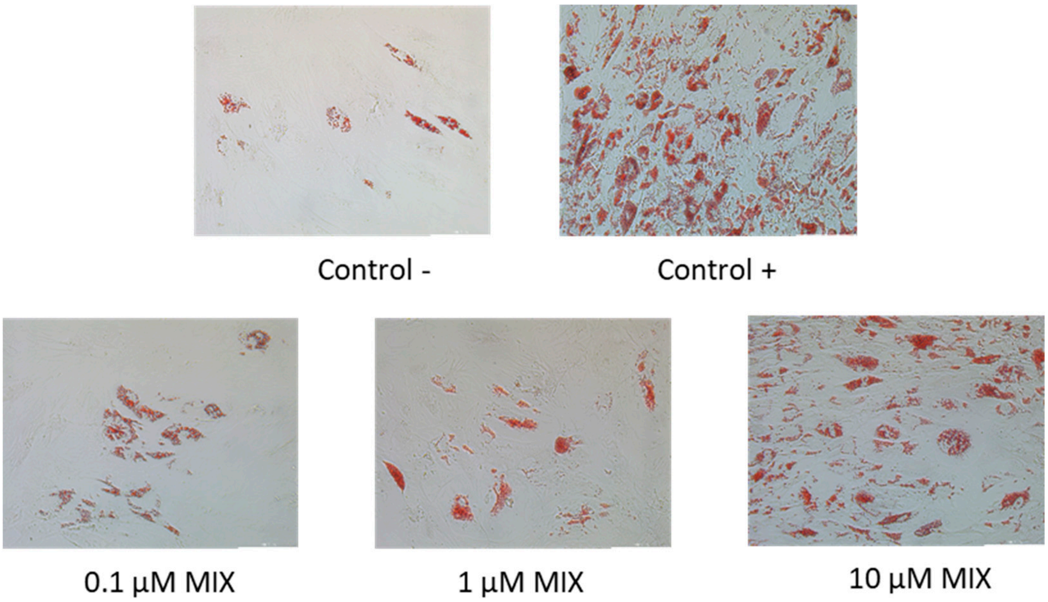

Figure S1. hASCs visualized by Oil Red O staining assay after 14 days of adipogenic differentiation. Human adipose derived stem cells were cultured in the presence of a mixture of three bisphenols (MIX: BPA, BPS and BPF) at different concentration (0.1, 1, or 10 µM MIX) and visualized at 20X under Leica DMi8 microscope (Leica Microsystems). hASCs, human adipose-derived stem cells.

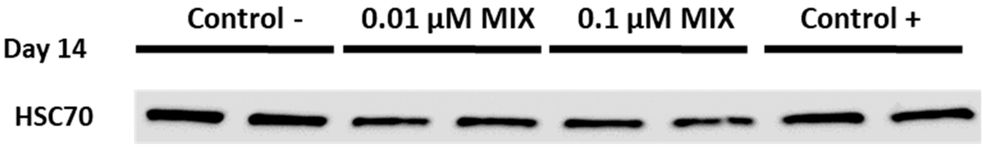

Figure S2. Western Blot of the control protein (HSC70) at the lowest concentrations of the mixture tested (0.01 and 0.1 µM). After 14 days of hASCs differentiation, protein levels of HSC70 were assessed to normalize the selected adipogenic markers. MIX, mixture of three bisphenols (BPA, BPF and BPS).

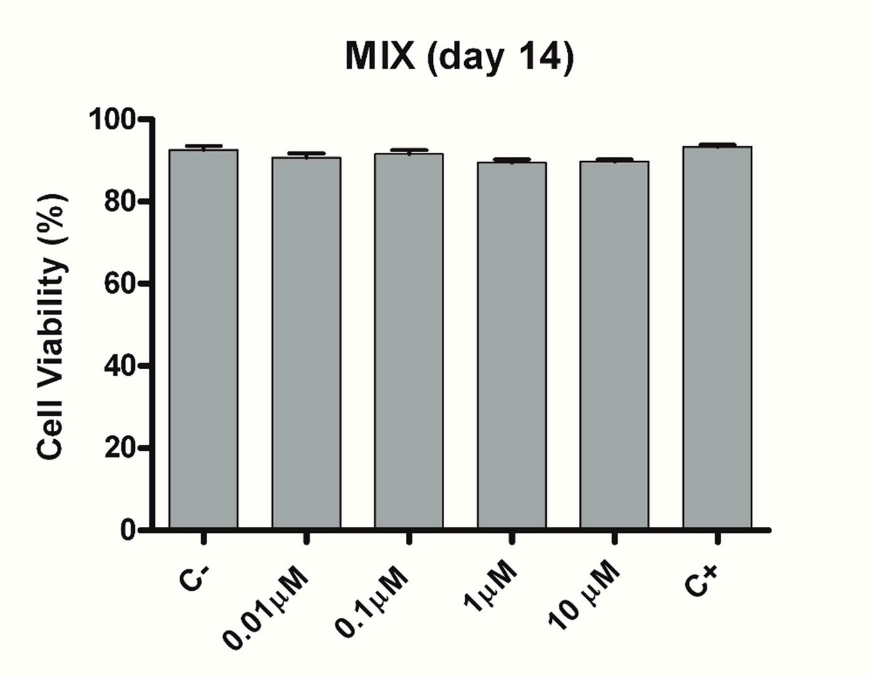

Figure S3. Effect of the mixture of three bisphenols (BPA, BPS, and BPF) on the viability of hASCs after 14 days of adipogenic differentiation. hASCs were differentiated in the presence of different concentrations of the mixture, and trypan blue assay was used to assess their viability. Cell viability (%) was expressed as means ± SEM from three independent experiments with multiple replicates for each experimental condition. BPA, bisphenol A; BPF, bisphenol F; BPS, bisphenol S; C−, negative control; C+, positive control; hASCs, human adipose-derived stem cells; MIX, mixture of three bisphenols (BPA, BPF and BPS); SEM, standard error of the mean.
